# Analysis of Exhaust Gas Content for Selected Biofuel-Powered Combustion Engines with Simultaneous Modification of Their Controllers

**DOI:** 10.3390/ma14247621

**Published:** 2021-12-10

**Authors:** Marietta Markiewicz, Michał Pająk, Łukasz Muślewski

**Affiliations:** 1 Faculty of Mechanical Engineering, Bydgoszcz University of Science and Technology, Alley of Professor S. Kaliskiego 7, 85-796 Bydgoszcz, Poland; lukasz.muslewski@utp.edu.pl; 2 Faculty of Mechanical Engineering, University of Technology and Humanities in Radom, Stasieckiego 54, 26-600 Radom, Poland; m.pajak@uthrad.pl

**Keywords:** combustion engine, biofuel, renewable energy sources, components of combustion engine exhaust gases, controllers of a vehicle computer

## Abstract

The use of renewable resources for powering self-ignition engines in European Union countries involves a high demand for renewable energy which is not accompanied by the development of its production infrastructure. The application of biofuel in vehicle powering is supposed to provide reductions in greenhouse gas emissions and an increase in the share of renewable energy resources in the total energy consumption. The study includes the analyses of power unit exhaust components, such as oxygen, carbon monoxide, nitric oxides, carbonizers, carbon dioxide and a quantity of exhaust particles contained in exhaust gases. Tests using an exhaust gas analyzer and a vapor analyzer were conducted. Three high-pressure engines, characterized by direct fuel injection, were tested. The vehicle computer software adjustments included increasing the fuel dose and the air load. Mixtures of diesel oil and fatty acid methyl esters were used in the tests. Based on the results, a statistical analysis was performed and an assessment model was developed to understand the functioning of the research objects fueled with these mixtures, with simultaneous software changes in the vehicle computers. On the basis of the conducted analysis, it was found that only 30% of fatty acid methyl ester additives to diesel oil reduced the performance parameters of the drive units.

## 1. Introduction

Nowadays, the protection of the planet environment and its natural resources is an important issue. The addition of biocomponents to diesel oil recommended by the European Union makes it possible to decrease the use of fossil fuels. Structural solutions of the combustion engine structure make it possible for engines to be modernized and adjusted for the application of plant oils. There are a few arguments in favor of biofuels. The first argument is related to the exhaustibility of petroleum-derived fuels; the second consists in relieving the market of petroleum-derived product delivery; and the third one regards the intensification of rural areas. Although the prognoses regarding the exhaustion of fossil fuels are more optimistic than before, there are still many social, economic and political factors that indicate the need to carry out tests on the suitability of plant oils to be used for combustion engine powering. Another argument in favor of using plant oils as engine fuel is CO_2_ emission reduction. The emission of toxic substances from transport is a significant problem, especially because, in the European Union countries, it was reported to be 0.4 g/km in 2019.

The basic fuel used in self-ignition engines is diesel oil. Due to the rising prices of petroleum-derived fuels and the exhaustion of natural resources, the idea of renewable fuels has been revived. Renewable fuels (of natural origin) include rape oil, sunflower oil, soya oil, peanut oil and animal fats [1]. Plant fuels (biofuels) need to undergo chemical processing to achieve physical–chemical properties similar to those of diesel oil. Due to technical, structural and economic factors, it is rape oil that finds the widest application [2,3]. Biogas from fruit, vegetables and meat production waste can also be used for powering self-ignition engines [4,5,6]. Alternative fuels that are produced from unfit-for-use food products are referred to as second-generation fuels. Another source of biofuel are microalgae (third-generation fuels), whose cultivation involves only CO_2_ and solar energy [4,7,8]. Numerous scientific and industrial centers deal with testing fatty acid methyl ester additives to diesel oil. The test results indicate that their physical–chemical properties are the most similar to those of a diesel oil.

**Figure 1:**
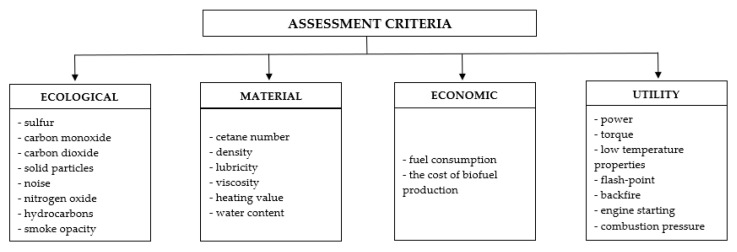
Criteria for assessment of transport means drive unit [16].

The literature provides the results of tests on engine operation efficiency, fuel consumption and the amounts of substances in exhaust gases, including solid particles. Measurements of the operation efficiency of engines powered with first-generation biofuel have already been performed by many scientific units. The results confirm changes in the power-unit performance parameters, including fuel density, increase in fuel flow resistance and reduction in engine power [9,10,11]. The results of the tests indicate an increase in fuel consumption when using mixtures of diesel oil and biocomponents for powering engines [12,13,14,15]. The quantities which describe a combustion engine performance (performance parameters) are indicators of its operation and their analysis provides information on its characteristics. The criteria for the assessment of self-ignition engines powered with mixtures of diesel oil and biocomponents are based on the information available in the literature [16,17,18,19,20,21]. Figure 1 shows their graphical interpretation.

The most frequently used indicators of a self-ignition engine operation assessment are power output, torque, fuel consumption, composition of exhaust gases and exhaust smoke. According to scientific publications, the use of biofuels for engine powering contributes to a decrease in the emission of carbon monoxides, hydrocarbons and solid particles [22,23,24,25,26,27,28] and an increase in nitric oxides by several percent, as compared to diesel oil [13]. These quantities are largely affected by the fuel physical–chemical properties, which are presented in Table 1. This study presents a comparison of the results for diesel oil, biodiesel and vegetable oil that had not been chemically treated for use as diesel fuel. However, the presented biodiesel comprised fatty acid methyl esters or vegetable oil that had been transesterified.

The study presents the results of fuel component tests and content of solid particles in exhaust gases of combustion engines powered with mixtures of diesel oil and fatty acid methyl esters. Moreover, the computer software of the analyzed power units was modified by changing the fuel dose and air load. The purpose of the tests was to check how the fuel mixture and reprogramming of the computer controllers affected the analyzed fuel component amounts. The study is also an attempt to demonstrate the content of fatty acid methyl esters added to diesel fuel from the point of view of the power-unit performance parameters.

## 2. Materials and Methods

**Figure 2:**
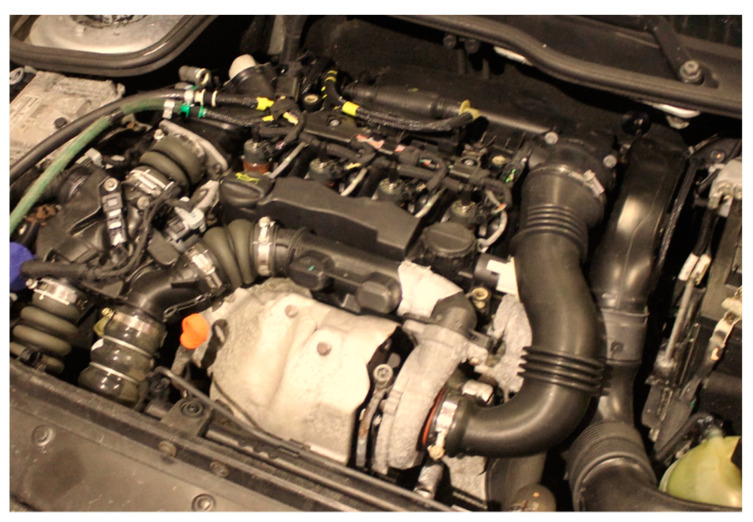
Combustion engine used in tests.

The tested objects were three self-ignition engines with identical technical parameters. The engines are widely used in car transport. They are mounted in passenger and delivery cars of different makes. The engines used for the tests were popular models that are found in vehicles. Three identical power units were used to provide reliable results. The tests covered engines with power output 81 kW, characterized by indirect fuel injection and a common rail fuel injection system. The tested engines were mounted in vehicles, thanks to which it was possible to simulate road conditions. An image of one of the analyzed engines, which was installed in a vehicle, is presented in Figure 2, whereas technical specifications of particular engines are presented in Table 2.

The research objects were modified by adjusting the fuel supply system which enabled noninvasive exchange of the fuel mixture. These changes covered the fuel supply system and involved the installation of an additional fuel tank. The modifications had no direct effect on the engine structure. The changes involved the installation of an external fuel tank while disconnecting the liquid flow from the vehicle internal tank. No additional filters or fuel pumps were fixed. The fuel supply system was connected from an additional tank directly to the engine. Standard filters suitable for a given engine model were added. The excess of fuel returned to the external tank through a return fuel tank. Each time after a mixture was exchanged, the engines worked for 10 min with the gears in neutral in order to remove the remains of the previous fuel from the fuel filter and the fuel supply system.

The research material was a ‘virgin’ diesel oil (ON) and fatty acid methyl esters of rape oil (RME). Diesel oil without a biocomponent additive was the virgin diesel oil used in the tests. The composition of the analyzed mixtures and their markings are presented in Table 3. Fatty acid methyl esters are plant oils that have been subjected to catalytic esterification. Such substances are commonly referred to as biocomponents. The fatty acid methyl esters of rape oil used in the tests were provided by domestic producers and the plants for their production were obtained from regional suppliers.

Based on the analysis of the literature and the authors’ own tests for various proportions of the tested mixtures, it was decided to use the mixtures with the composition presented in Table 4. First, selected properties of the tested mixtures were determined, such as viscosity, calorific value, heat of combustion and cetane number. The obtained results are presented in Table 4. The table shows the mean values for 30 measurements.

**Figure 3:**
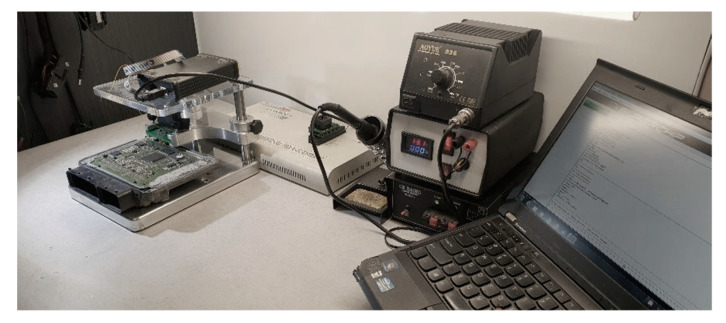
Stand for modification of vehicle computer software.

The engines were also modified by adjusting the electronic system through modification of computer software. The introduction of computer software changes was performed to find out whether and how the number of exhaust components and the number of solid particles emitted to the environment changed. Due to the specificity of the electronic system, the software changes required dismantling the deck computer and mounting it on the modification stand, as shown in Figure 3.

Software modifications were introduced according to an earlier prepared schedule. They involved increasing the dose of fuel and the air load. The tests were performed for four fuel injection controller settings, which are presented in Table 5.

**Figure 4:**
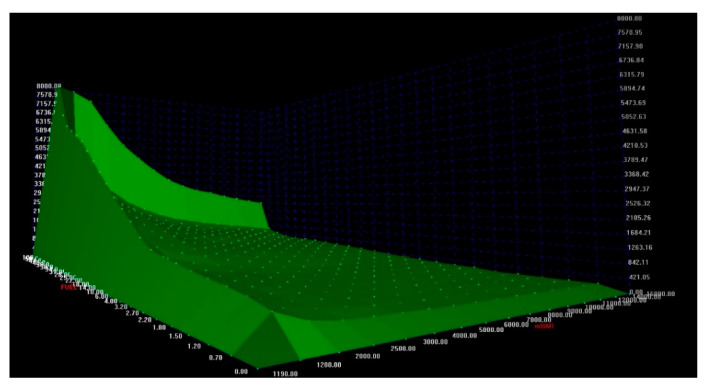
Stand for modification of vehicle computer software.

Each fuel injection setting required the generation of a new fuel injection characteristic. Four characteristics were created for each fuel dose and fuel injection pressure. One of the characteristics is presented in Figure 4.

The experiment involved measuring the pressure of the exhaust gas component concentration and the number of solid particles. The measurement of the exhaust gas concentration was performed by means of an MGT-5 exhaust gas analyzer. The analysis of the exhaust gases enabled determination of the amounts of exhaust substances which were discharged into the environment in the form of exhaust gases. The described device was used for the measurement of the toxicity degree of a self-injection engine exhaust and was designed for the determination of the values of compounds such as hydrocarbons (HC), carbon dioxide (CO_2_), nitric oxides (NO_2_) and carbon oxide (CO). The analyzer can also be used for determination of exhaust gas components which are not environmentally harmful including oxygen (O_2_), and a coefficient of air excess ƛ. The measurement of solid particles with dimensions exceeding 100 mm was performed by means of an MPM-4 analyzer. An optical method involving measuring the intensity of a light beam passing through the stream of exhaust gases was used for the measurement of the mass of solid particles contained in the exhaust gases.

The tests were conducted with the use of a load-bearing chassis dynamometer with an eddy current brake which allowed us to simulate road conditions and apply proper loads to the vehicles.

## 3. Analysis of Test Results

### 3.1. Statistical Analyses of the Test Results

**Figure 5:**
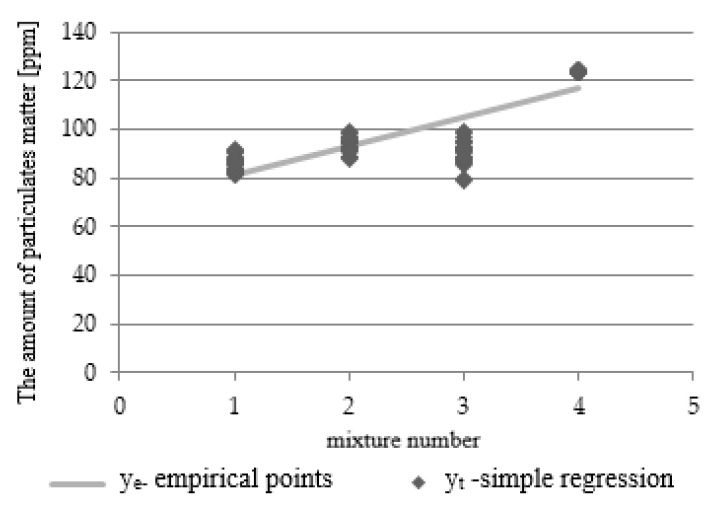
Diagram of linear regression for the analyzed parameter.

A linear multi-factor regression equation was developed to assess the dependence of the power unit performance parameter values Y for fuel mixtures, dependent on the fuel injection modification X. Six performance parameters (fuel components and solid particles) were tested for the four settings of combustion engine computer software and four fuel mixtures. The test results were averaged for the three analyzed power units. Since numerous calculations had to be performed, the study includes an exemplary regression equation for the ‘solid particles’ parameter based on the vehicle computer software settings. The graphic interpretation of the obtained results is shown in Figure 5 as follows: a chart shows the empirical points, while a graphical chart is depicted as a determinant of theoretical parameters.

The values of the estimators were determined by means of the method of the smallest squares. The differences between the measurement values, their mean values and the values of the defined functions were also calculated. Based on this, a straight of regression was estimated, which, for setting III, takes the following form:

y = 0.1431x + 88.015(1)

The correlation coefficient for an increased regression equation was r = 0.5151. Testing the H_0_: a = 0 hypothesis yielded *p* < 0.0001, which means that the analyzed dependence was statistically significant.

The distribution of the obtained results was verified prior to the analysis of linear regression performed by means of X^2^ Pearson and λ Kołmogorov consistency tests. The distribution was found to be normal. A variance analysis was also performed. The test involved comparing the mean values for the four mixtures of diesel oil and fatty acid methyl esters. Four tests were conducted for each engine software setting (I, II, III and IV) to investigate relevant parameters of the analyzed power units. Equal mean values of all the mixtures were accepted to be the zero hypothesis.

### 3.2. Figures, Tables and Schemes

This study takes into consideration ecological parameters, evaluated in terms of environmental impact. These parameters were chosen due to their environmental impact and functioning of combustion engines, which are powered with mixtures of diesel oil and fatty acid methyl esters. The physical–chemical properties of these mixtures, such as calorific value, viscosity and cetane number, were also tested.

The aim of the tests was to compare power units powered with different mixtures of virgin diesel oil and fatty acid methyl esters. The assessment of the power units was performed on the basis of their significant characteristics [29]. The assessment covered the distinguished ecological parameters and those described earlier. It was a normalized comparative assessment which referred to the accepted point of reference. In the analyzed case, the values of the characteristics describing the analyzed parameters could be lower or higher than the values of characteristics that represented the point of reference.

In the presented model, variable X means unidimensional vector (tested parameters of power units), which was accepted to be a random variable. Vectors (X_1_ ÷ X_7_) represent an assessment of power units powered with different fuel mixtures. The considered vector takes the following form:

X_i_ = <X_1_, X_2_, X_3_, X_4_, X_5_, X_6_, X_7_> (2)

where the vector components include the following:

X_1_—particulates contained in exhaust gases;
X_2_—carbon monoxide;
X_3_—carbon dioxide;
X_4_—hydrocarbons;
X_5_—oxygen;
X_6_—nitric oxides;
X_7_—air excess coefficient.

The assessment process was performed with the use of a multi criteria optimization analysis (MOA) [30]. The multi criteria optimization analysis enabled us to compare the same parameters of power units powered with different fuel mixtures. Although the characteristics of the analyzed mixtures are hardly comparable, they had a significant impact on the research object’s functioning and the natural environment. The analysis included different variants of the environmental criterion. The variants evaluated within a given criterion were arranged in a specified order. The quality of particular variants was also defined by assigning appropriate results to them. The application of this method enabled the determination of a qualitative criterion, a system of weights for particular variants and the performance of the whole assessment for the environmental criterion, as well as its interpretation. The method of analytical hierarchy process (AHP) was used during the MOA analysis for the determination of the weights for each variant [31,32].

A random variable was defined for the research object as follows:

Z_x_ = α_1_X_1_ + α_2_X_2_ + α_3_X_3_ + α_4_X_4_ + α_5_X_5_ + α_6_X_6_ + α_7_X_7_ (3)

where α refers to the values of the weights for particular parameters.

The developed assessment system includes seven criteria defined on the basis of the power-unit parameters. The mean value of 30 measurements was the assessed value. The criteria argument scopes were limited by the highest and the lowest values provided by the tests. All the criteria were accepted in the form of MINSIMP, which means that the lowest values, consistent with the lowest emission of a given substance, were found to be the most optimal. The weights of particular criteria were defined during the tests and are presented in Table 6.

A comparison of the tested mixtures of diesel oil and fatty acid methyl esters based on the evaluation of particular variants allowed us to refer the parameter values of to a ‘virgin’ diesel oil that, in this study, was assumed to be the point of reference. The performed assessment is expressed as an arithmetic mean, this being the most effective, unburdened estimator of an unknown expected value [33], and is presented in Table 7 with a division into particular variants.

**Figure 6:**
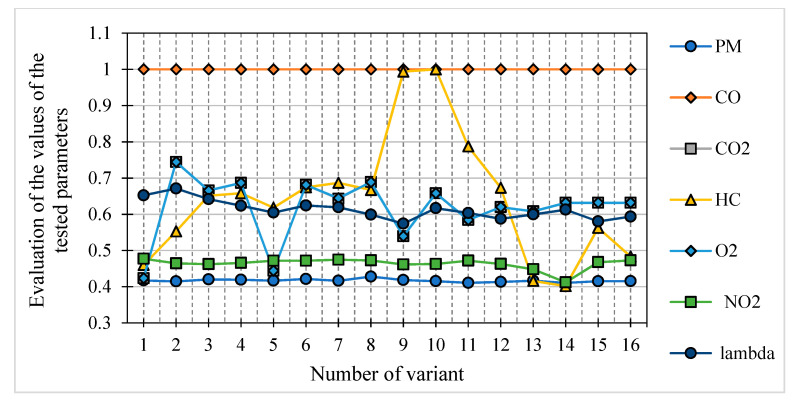
Assessment of the tested parameter values for particular fuel mixtures.

The results presented in the table present the ratings for individual operational parameters and sixteen variants of the mixture–controller setting. The graphic interpretation of these results is shown in Figure 6.

The measurements performed for four mixtures of diesel oil with fatty acid methyl esters and four computer software modifications of the analyzed vehicles allowed us to obtain 16 variants and provide a complete assessment for all the analyzed criteria, which is presented in Table 8.

**Figure 7:**
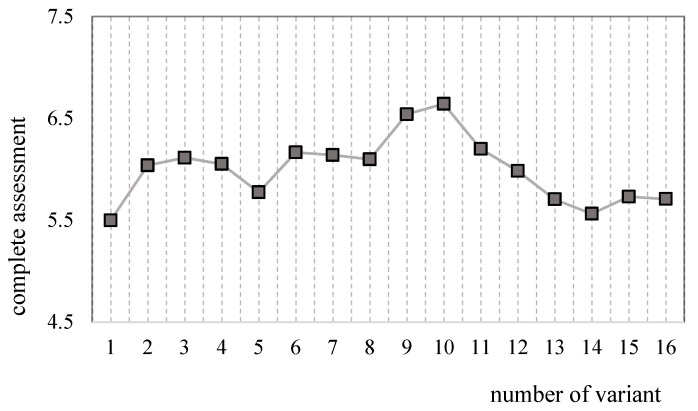
Complete assessment for particular variants.

The graphical interpretation of the obtained scores for individual variants is shown in Figure 7.

In terms of the assessment criteria, variant number 10, that is, a BIO30 mixture with an increased fuel dose of 4% and air load of 50 hPa, which was 6.6417, was found to be the best one. The use of BIO30 fuel to power the engine enabled to obtain the best assessment results for all computer software adjustments. By using the virgin oil-powered engine to be a reference point, it was found that the use of the BIO10 and BIO30 mixtures improved the performance quality of the analyzed power units in terms of the accepted criteria.

It needs to be emphasized that, by referring the assessment result scatter to a possibly higher scatter of assessment results, which can be obtained using this method (from 4 to 10), an exchange of a mixture involves, approximately, a 19% change in the tested object performance quality. Hence, this issue has a significant impact on the rationalization of the process of self-ignition combustion engine operation.

The test results allowed us to define the vector components quantities. The determination of these components enabled us to perform the geometric interpretation of the parameter mean values. For the purpose of transparency and unequivocality of the results, their values were normalized into the interval <0 ÷ 10> for the set of the analyzed parameters <0 ÷ 10>, using the following dependency:

(4) $$10\times \frac{\left(X_{i}-X_{\min}\right)}{\left(X_{\max}-X_{\min}\right)}$$

Variability intervals were also determined for the analyzed parameter set. The vector components were analyzed for the four tested fuel mixtures with averaged modifications of the fuel injection controller. The minimal values obtained from experimental tests were accepted to be the most desired result for the tested parameters. The normalized results of the particular vector components are presented in Table 9.

**Figure 8:**
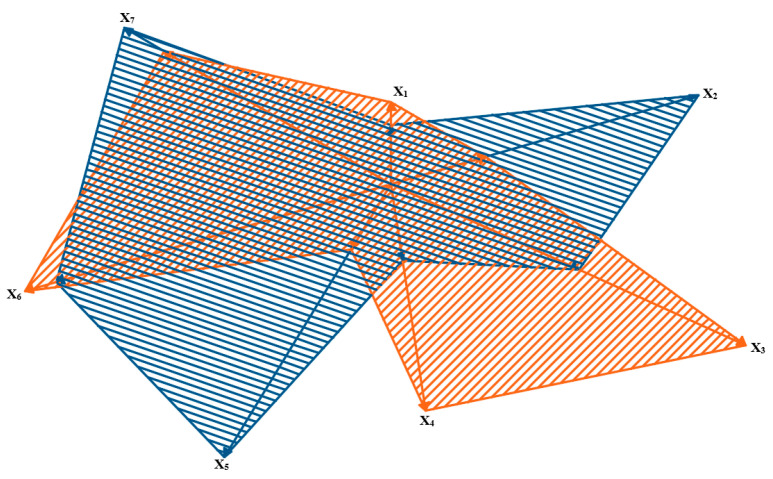
Graphic interpretation of comparative analysis of results of particular component parameters for BIO 10 and ‘virgin’ diesel oil.

**Figure 9:**
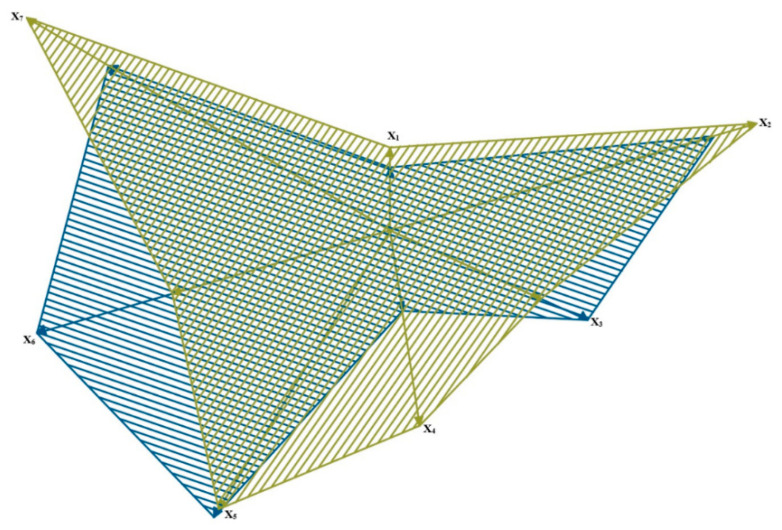
Graphic interpretation of comparative analysis of results for particular parameters of components for BIO 30 and ‘virgin’ diesel oil.

**Figure 10:**
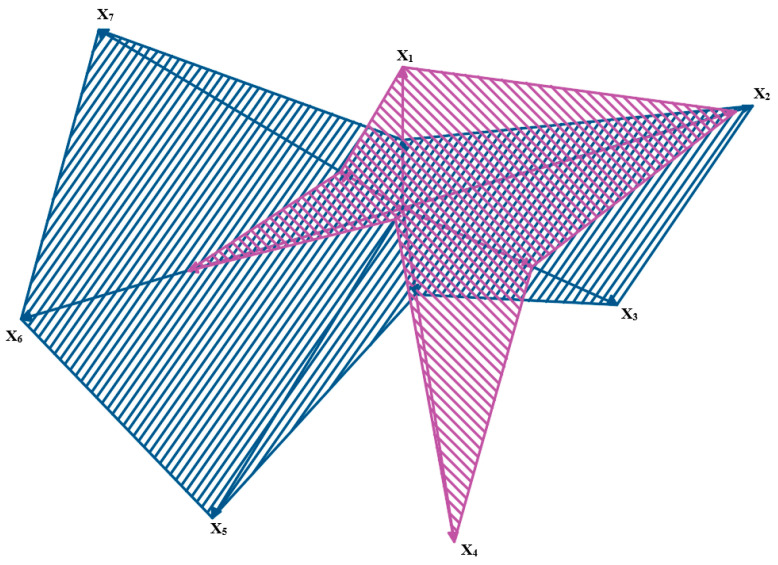
Graphic interpretation of comparative analysis of results for particular parameters of components for BIO 50 and ‘pure’ diesel oil.

The results are presented in a normalized form in such a way that number 0 means the lowest score, whereas 10 means the highest one. Thus, the minimal score is the best one for the considered power unit parameters. The results obtained for the analyzed parameters in particular variants (fuel mixtures) are presented in a geometric interpretation in Figure 8, Figure 9 and Figure 10. The blue color was used for marking ‘virgin’ diesel oil, which, in the analyzed case, was the point of reference for the remaining fuel mixtures. The other colors (orange, green and violet) were used to mark the tested mixtures of diesel oil and fatty acid methyl esters—respectively, BIO10, BIO30 and BIO50. The analyzed power units are the vector components in a graphic interpretation.

The above-presented schemes show that the lowest parameter values were found for the BIO50 mixture (except for the parameters of solid particles and hydrocarbons). For the BIO10 mixture, the analyzed parameters had higher values than the reference point, that is, ‘virgin’ diesel oil. A drop in the value of certain parameters was observable for BIO30, these being carbon dioxide and nitric oxides. An analysis of the data showed that only a 30% additive of fatty acid methyl esters to diesel oil decreased the number of components in exhaust gases which can have a negative influence on the natural environment. It is the optimal amount of the biocomponent that should be added to diesel fuel in order to lower the engine parameters.

**Figure 11:**
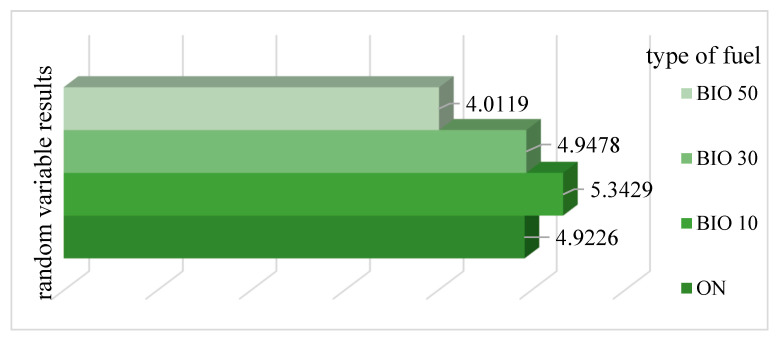
Standardized results of random variable.

The mean values of the results provided for the three analyzed research objects allowed us to define the quantity of random variable Z_x_. The standardized results of the random variable are presented in Figure 11.

The values of the random variable Z_x_ for particular fuel mixtures (ON, BIO10, BIO30 and BIO50) reflect the sum of the vector component products and assign them weights. The ON mixture (‘virgin’ diesel oil) was the point of reference. As can be seen in the chart, the random variable determined for the BIO10 mixture (90% of diesel oil and 10% of fatty acid methyl esters) and BIO30 mixture (70% of diesel oil and 30% of fatty acid methyl esters) is higher than that of the point of reference. The random variable of the BIO50 mixture is lower by approximately 18% than that of the point of reference. The obtained results indicate a beneficial effect of the BIO50 mixture.

### 3.3. Verification of the Proposed Assessment Model

One of the problems of the assessment process is the determination of only those parameters which significantly affect the assessment from the point of view of the analyzed parameter set; the selected parameters provide the basis for further analyses. A set of seven parameters was distinguished in the proposed model. To verify this model, the method of mean fuzzy charts was used. The application of elements of fuzzy logic allowed us to correct the redundancy of the parameter set and to determine the significance of these parameters. In order to analyze real data, the value established for the cross-section by measuring points was fuzzified. The belonging of the measuring point to a given cross-section was rendered in the form of a Gauss function. Original software was used to perform the analyses. The value of the membership function coefficient range was 20%. The number of fuzzy cross-sections was established to be seven and a method was developed for the scatter calculation as a mean square value. The value 0.01 was accepted to be the significance limit [33,34]. By providing all the analyzed performance parameters with identical parameters, it was found which ones were the most significant from the point of view of the experiment. The test result mean values of the analyzed fuel mixtures performance parameters and fuel injection controller settings are presented in Table 10.

**Figure 12:**
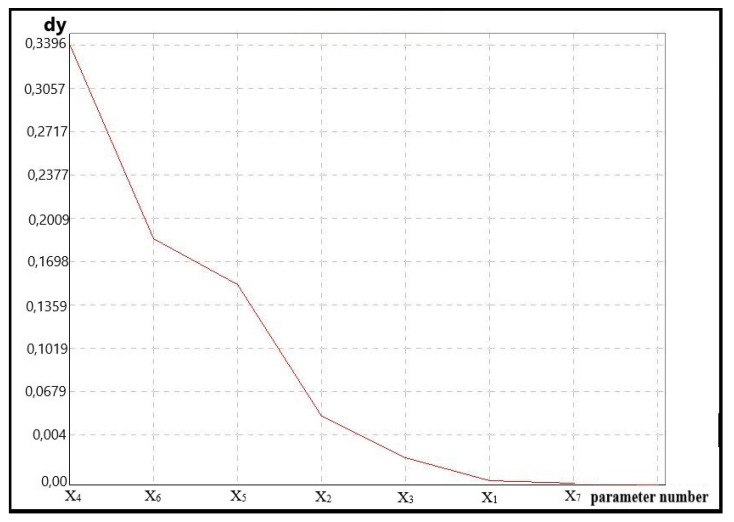
Spectral form.

The results achieved in this way were analyzed through the determination of fuzzy means to identify the change in sensitive parameters. Figure 12 shows a gradient form of fuzzy means. The analyzed parameters are demonstrated in a decreasing manner according to the values of their scattering.

Based on data analysis, it can be accepted that the parameters whose scatter value is lower than 0.04 were not sensitive to the changes introduced in engine computer software and their values did not undergo statistically significant change depending on the fuel mixture used. The values of scatter obtained for particular parameters, defining their sensitivity to the fuel mixture change and the power unit computer software, are demonstrated in Table 11.

**Figure 13:**
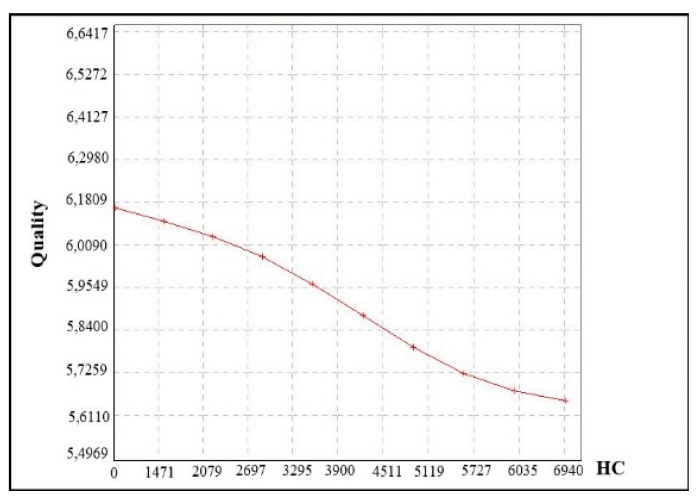
A chart of fuzzy means for sensitive data on the basis of parameter X_4_.

**Figure 14:**
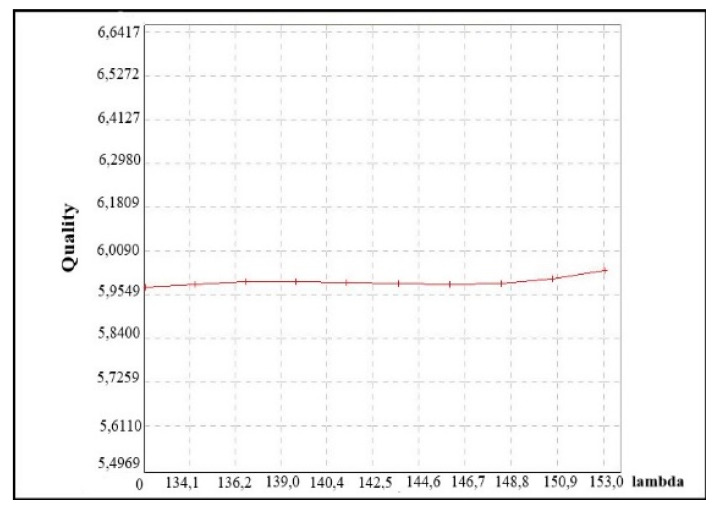
A chart of fuzzy means for little sensitive data on the basis of parameter X_7_.

The test results make it possible to find out which of the analyzed parameters was sensitive to the changes and whether it can be omitted in further research. Exemplary charts of the most sensitive parameter X_4_ and little sensitive X_7_ are presented in Figure 13 and Figure 14.

## 4. Discussion

The need to reduce the emission of harmful fuel components from self-ignition engines raises interest in research on new power supply solutions. The test results prove that the use of mixtures of diesel oil and fatty acid methyl esters contributes to the reduction in the amount of fuel components emitted into the environment, which has also been confirmed by many authors [35,36,37,38,39]. The test results involve different settings of power-unit computer software. The literature does not provide data on the subject of fuel injection controller adjustment that would enable us to match a given setting to obtain optimal performance of the power unit powered with a mixture of diesel oil and fatty acid methyl ester. Adjustments of vehicle computer software would allow us to decrease the number of exhaust gas components. The most important aspect of the study is the assessment of engine performance depending on the fuel mixture composition. This was possible by obtaining the measurements for fatty acids of diesel oil, those of the ester service and service updates for 16 updates and the evaluation of methyl services. The conducted assessment shows the last grade of the coal assessment and the lowest grade for the assessment performed. The assessment of the significant it is the most unstable, its results ranging around 1, as well as being equal for the equation that contains lambda. The ratings for the experiments vary between 0.4 and 0.7. Location data should be analyzed in two ways; in addition to the analysis of the operational parameters, the verification of the variant sample should be carried out as follows: the first assessment should be performed for the second, eighth and tenth variants, while the lowest assessment should be performed for the first, thirteenth and fourteenth variants. From the analysis of the evaluation scores, out of all them, the evaluation scores of the controls for the tenth variant were lower than the previous one. The article also presents the verification of individual performance parameters in terms of assessment. On the basis of the control performed, changes to the settings were made.

## 5. Conclusions

An analysis of the values of particular parameters, based on the random variable defined for each research object, showed that the best results were obtained for the BIO10 mixture and the worst ones for BIO50. It can be observed that the best mixture was the one with 70% of diesel oil and 30% of fatty acid methyl esters. Changes in the analyzed parameter properties were represented in a vector-based form which enabled the simultaneous analysis of their changes. From the point of view of exhaustibility of petroleum, the use of alternative solutions for powering combustion engines is justified. The application of fatty acid methyl esters makes it possible to reduce the consumption of diesel oil. According to the established 10-point rating scale, the assessment of the impact of the fuel injection controller settings on the values of the performance parameters of engines fed with mixtures of fatty acid methyl esters and diesel oil is as follows: In the case of setting I for mixture III, the assessment was 8 and the difference between the highest and the lowest rating for individual mixes was about 16%, while, in the case of setting II for mix V, the assessment was 8 and the difference between the highest and the lowest rating for individual mixtures was about 36%. In the case of setting III for mixture V, the assessment was 8.2 and the difference between the highest and the lowest grade for individual mixtures was about 6%, whereas, in the case of setting the IV for mixture V, the assessment was 8.3 and the difference between the highest and the lowest grade for individual mixtures was about 13%. Finally, in the case of setting V for mixture V, the assessment was 8 and the difference between the highest and the lowest grade for individual mixtures was about 17%. This means that there is a correlation between the fuel mixture composition and the fuel injection controller settings. The applied method of fuzzy diagrams is an important tool to be used for the assessment of the influence of fatty acid methyl esters addition to diesel oil on transport means power-unit functioning, including the optimal setting of the fuel injection controller. The test results confirm advisability of using alternative fuels with appropriate component proportions and adequate settings of the power unit fuel injection controller.

## Figures and Tables

**Table 1:** Comparison of fuel physical chemical properties.

Property	Diesel Oil	Biodiesel	Plant Oil
Viscosity (mm^2^ × s^−1^)	2.0–4.5	3.5–5.5	7.2
Sulfur content (mg × kg^−1^)	≤35	≤10	no
Density (g × cm^−3^)	0.82–0.45	0.86–0.90	0.88
Cetane number	≥51	≥47	>40
Calorific value (MJ/kg)	43	41	37.6
Ignition point (°C)	≥55	≥101	≥220

**Table 2:** Technical specifications of combustion engines used in the tests.

Type of Engine	With Self-Ignition
Kind of fuel	Diesel oil
Power output of engine	81 kW
Engine cubic capacity	1560 cm^3^
Maximal torque	240 Nm
Number of cylinders	4
Diameter of cylinder	73 mm
Piston stroke	88.3 mm
Number of valves	16
Particulate filter	none
Compression ratio	16.0:1

**Table 3:** Composition of fuel mixtures.

Composition of Fuel Mixture	Symbol of Mixture
“virgin” diesel oil	ON
90% diesel oil, 10% fatty acid methyl esters	BIO10
70% diesel oil, 30% fatty acid methyl esters	BIO30
50% diesel oil, 50% fatty acid methyl esters	BIO50

**Table 4:** Properties of the tested mixtures.

Properties	ON	BIO10	BIO30	BIO50
Viscosity	6.46	6.26	7.8	8.56
Calorific value (J/g)	43,097.33	43,199.33	41,959.00	40,590.00
Heat of combustion (J/g)	44,277.33	44,379.00	43,139.00	41,770.00
Cetane number (mPa × s)	53.33	54.94	55.92	58.40

**Table 5:** Composition of fuel mixtures.

Modifications of Computer Software	Marking of Setting
Factory settings	I
Fuel dose increased by 2% and air load increased by 50 hPa	II
Fuel dose increased by 4% and air load increased by 50 hPa	III
Fuel dose increased by 6% and air load increased by 50 hPa	IV

**Table 6:** Values of weights for particular parameters.

Marking	Weight
α_1_	0.19365
α_2_	0.16125
α_3_	0.16125
α_4_	0.16125
α_5_	0.1291
α_6_	0.16125
α_7_	0.03225

**Table 7:** Assessment of the tested parameter values for particular fuel mixtures.

Number of Variant	Symbol of Mixture	Number of Setting	PM	CO	CO_2_	HC	O_2_	NO_2_	Lambda
1	ON	1	04174	1	0.4234	0.4598	0.4234	0.4771	0.6522
2	ON	2	0.4147	1	0.7443	0.5527	0.7443	0.4645	0.6710
3	ON	3	0.4202	1	0.6657	0.6512	0.6657	0.4622	0.6418
4	ON	4	0.4193	1	0.6865	0.6581	0.6865	0.4658	0.6236
5	BIO10	1	0.4167	1	0.4439	0.6181	0.4439	0.4717	0.6047
6	BIO10	2	0.4214	1	0.6810	0.6744	0.6810	0.4717	0.6242
7	BIO10	3	0.4164	1	0.6429	0.6868	0.6443	0.4745	0.6193
8	BIO10	4	0.4280	1	0.6887	0.6671	0.6887	0.4726	0.5990
9	BIO30	1	0.4185	1	0.5393	0.9937	0.5393	0.4614	0.5741
10	BIO30	2	0.4153	1	0.6580	1	0.6579	0.4627	0.6169
11	BIO30	3	0.4107	1	0.5843	0.7872	0.5843	0.4720	0.6035
12	BIO30	4	0.4132	1	0.6196	0.6732	0.6196	04630	0.5875
13	BIO50	1	0.4168	1	0.6081	0.4161	0.6081	0.4482	0.5996
14	BIO50	2	0.4106	1	0.6317	0.4018	0.6317	0.4121	0.6126
15	BIO50	3	0.4152	1	0.6317	0.5624	0.6317	0.4678	0.5802
16	BIO50	4	0.4153	1	0.6317	0.4834	0.6317	0.4726	0.5935

**Table 8:** Complete assessment for particular variants.

Number of Variant	Symbol of Mixture	Number of Setting	Complete Assessment
1	ON	1	5.4968
2	ON	2	6.0378
3	ON	3	6.1121
4	ON	4	6.0511
5	BIO10	1	5.7727
6	BIO10	2	6.1647
7	BIO10	3	6.1391
8	BIO10	4	6.0970
9	BIO30	1	6.5388
10	BIO30	2	6.6417
11	BIO30	3	6.1993
12	BIO30	4	5.9829
13	BIO50	1	5.7043
14	BIO50	2	5.5627
15	BIO50	3	5.7302
16	BIO50	4	5.7067

**Table 9:** Assessment of the tested parameter values for respective fuel mixtures.

Vector Components	Symbol of Mixture			
	ON	BIO10	BIO30	BIO50
X_1_	1.361	2.249	1.801	2.819
X_2_	7.287	8.867	8.267	6.933
X_3_	4.701	5.211	3.589	2.844
X_4_	1.759	1.745	4.275	6.755
X_5_	7.281	8.695	7.081	0.265
X_6_	7.951	6	4.9	4.467
X_7_	7.052	8.244	9.104	1.433

**Table 10:** Assessment of mean values for the accepted variants.

Symbol of Mixture	Number of Setting	Particulates	Carbon Monoxide	Carbon Dioxide	Hydrocarbons	Oxygen	Nitric Oxides	Excess Air Coefficient
ON	I	9400	13	1196	5147	1727	22,723	1355
	II	9593	10	1214	3083	581	25,040	1324
	III	8667	6	1178	1953	684	25,460	1372
	IV	12,890	6	1184	1900	646	24,813	1402
BIO10	I	8573	16	1199	2210	1578	23,717	1433
	II	9370	4	1170	1773	656	23,723	1427
	III	9067	8	1203	1677	723	23,200	1409
	IV	12,392	2	1127	1830	642	23,563	1444
BIO30	I	7503	7	1189	903	1025	23,777	1526
	II	8997	8	1210	863	698	25,367	1413
	III	9323	12	1240	1237	861	23,663	1435
	IV	12,035	8	1224	1783	768	25,310	1482
BIO50	I	6643	6	1200	6500	789	28,040	1442
	II	8567	9	1241	6943	746	34,683	1420
	III	9437	11	1211	5067	746	24,077	1506
	IV	12,105	4	1231	4413	746	23,547	1462

**Table 11:** Values of fuzzy mean scatter for the tested parameters.

Marking of Parameter	Value of Fuzzy Mean Scatter
X_1_	0.0032
X_2_	0.0491
X_3_	0.0210
X_4_	0.3396
X_5_	0.1524
X_6_	0.1886
X_7_	0.0014
